# The large *Gunnera’s (G*. *tinctoria* and *G*. *manicata)* in Europe in relation to EU regulation 1143/2014

**DOI:** 10.1371/journal.pone.0284665

**Published:** 2023-04-20

**Authors:** Johan L. C. H. van Valkenburg, Bruce A. Osborne, Marcel Westenberg

**Affiliations:** 1 Netherlands Institute for Vectors, Invasive Plants and Plant Health, NVWA, Wageningen, The Netherlands; 2 UCD School of Agriculture and Food Science, University College Dublin, Dublin, Ireland; 3 UCD Earth Institute, University College Dublin, Dublin, Ireland; University of Palermo, ITALY

## Abstract

Incorrect labelling of plants in the horticultural trade and misidentification is widespread. For the inspection services of the EU member states, correct identification of *G*. *tinctoria* has become important since the species was added to the List of Union concern in accordance with EU regulation 1143/2014 in August 2017. In the horticultural trade *Gunnera* plants are generally of modest dimensions and rarely flowering, so that the major distinguishing morphological characters for the identification of the two large species, *G*. *tinctoria* and *G*. *manicata*, are missing. As *G*. *tinctoria* is included in the EU regulation, its trade is prohibited, although the closely related species, *G*. *manicata* is not included on the list. Given that it is often difficult to distinguish between these two large herbaceous species using morphological attributes we used standard chloroplast DNA barcode markers, supplemented at a later stage by ITS markers. Plant material of putative *G*. *tinctoria* or *G*. *manicata* was obtained from the native and introduced range, both from “wild” sources, botanical gardens, and the horticultural trade. In western Europe plants circulating in the horticultural trade turned out to be predominantly *G*. *tinctoria*, with only one plant in cultivation identified as true *G*. *manicata* and the *G*. *manicata* found in botanical gardens was a hybrid recently described as *G*. *x cryptica*.

## Introduction

Incorrect labelling of plants in the horticultural trade and misidentification is widespread and may be caused by negligence or wilful disrespect of regulations [1–5]. Mislabelling may consist of simple misspelling of names or considering a variety as a true species, using synonyms or just preferring a name that sounds nice or a name that customers are familiar with. The latter two cases are indeed considered intentional misidentification of the plant in the horticultural trade. If mislabelled species are not regulated or do not pose a threat to the natural environment, only the customer should be affected (e.g. by not having the plant that they wished for). However, if the plant species is a potential threat to the environment or is regulated, the problem may be much more serious [6].

*Gunnera* L. is the single genus in the family Gunneraceae. According to Plants of the World online it comprises 63 species. Other authors sometimes mention it to be represented by 30–40 species [e.g. 7], mostly distributed in the Southern Hemisphere. The subgenus Panke to which the large perennial species found in European gardens belong comprises some 20 species found in Central and South America and Hawaii. The species of subgenus Panke are unique in having large, triangular scales between the leaves on the rhizomes and are characterised by rosettes of huge palmate leaves that account for their aesthetic value [8].

Despite questions about the nomenclature for the proper naming of *Gunnera manicata* Linden ex André and *G*. *tinctoria* (Molina) Mirb [9–11], the common opinion is that two kinds of large *Gunnera’s* predominate in western Europe. One of them is an invasive species and included in EU regulation 1143/2014 and named *Gunnera tinctoria* [12]. In Europe this species can form extensive long-lived naturalised populations under the high rainfall, high humidity conditions found in parts of Ireland, Portugal and the British Isles, which rarely experience extreme low temperatures [12]. Outside of Europe naturalised populations can be found under similar environmental conditions in the United States and in New Zealand, where invasive populations can also be found [13].

In the horticultural trade the typical *Gunnera* plants used are of modest dimensions and rarely flowering, so that the major distinguishing characters that might be used to distinguish between the two species are missing. It is also clear that it is not easy to distinguish between *G*. *tinctoria* and *G*. *manicata* based on morphological attributes alone [9, 12]. As *G*. *tinctoria* is included in the EU regulation, the trade of this species is prohibited, necessitating the use of other than macromorphological characteristics to clearly distinguish between the two species. The approach used in this study was to use standard chloroplast DNA barcode markers supplemented later with ITS markers to successfully distinguish between the two species.

## Material and methods

### Plant material

The plant material used in this study is shown in Table 1.

**Table 1:** Material used in this study.

Species (collected as)	Species^1^ (identified as)	Year	Voucher	Origin^2^	NCBI accessions (rbcL–trnH-psbA—ITS)
*G*. *manicata*	*G*. *manicata x G*. *tinctoria*	2017	WAG: Wieringa 9039	Netherlands: Hortus botanicus Leiden C	OQ241885—OQ241864—OQ222141
*G*. *tinctoria*	*G*. *tinctoria*	2017	WAG: Wieringa 9042	Netherlands: Hortus botanicus Leiden C	OQ241884—OQ241863—OQ222140
*G*. *tinctoria*	*G*. *tinctoria*	2018	WAGPD: Valkenburg 3913	New Zealand W	OQ241883—OQ241862—OQ222139
*G*. *tinctoria*	*G*. *tinctoria*	2018	WAGPD: Valkenburg 3915	New Zealand W	OQ241882—OQ241861—OQ222138
*G*. *manicata*	*G*. *manicata x tinctoria*	2018	WAGPD: Valkenburg 3928	New Zealand C	OQ241881—OQ241860—OQ222137
*G*. *manicata*	*G*. *tinctoria x manicata*	2018	WAGPD: Valkenburg 3948	Netherlands T	OQ241880—OQ241859—OQ222136
*G*. *manicata*	*G*. *tinctoria*	2018	WAGPD: Valkenburg 3949	Netherlands T	OQ241879—OQ241858—OQ222135
*G*. *tinctoria*	*G*. *tinctoria*	2018	WAGPD: Osborne s.n.	Ireland W	OQ241878—OQ241857—OQ222134
*G*. *manicata* (assumption)	*G*. *manicata*	2018	WAGPD: Valkenburg 4433	Sweden: Stockholm, Stockholm University C	OQ241876—OQ241855—OQ222132
*G*. *manicata*	*G*. *manicata x tinctoria*	2018	WAGPD: WAG60452354	Belgium: Meise, Meise Botanic Garden C	OQ241886—OQ241865—OQ222142
*G*. *manicata*	*G*. *manicata x tinctoria*	2018	WAGPD: Valkenburg 3973	France: Paris, Bois de Vincennes C	OQ241877—OQ241856—OQ222133
*G*. *manicata*	*G*. *tinctoria*	2019	WAGPD: Valkenburg 4432	Portugal: Azores T	OQ241875—OQ241854—OQ222131
*G*. *tinctoria*	*G*. *tinctoria*	2019	NPPO-NL ref. 6145796	Portugal, Azores, Sâo Miguel W	OQ241867—OQ241846—OQ222123
*G*. *tinctoria*	*G*. *tinctoria*	2019	NPPO-NL ref. 6145809	Portugal, Azores, Sâo Miguel W	OQ241868—OQ241847—OQ222124
*G*. *tinctoria*	*G*. *tinctoria*	2019	NPPO-NL ref. 6145817	Portugal, Azores, Sâo Miguel C	OQ241869—OQ241848—OQ222125
*G*. *tinctoria*	*G*. *tinctoria*	2019	NPPO-NL ref. 6145825	Portugal, Azores, Sâo Miguel W	OQ241870—OQ241849—OQ222126
*G*. *tinctoria*	*G*. *tinctoria*	2019	NPPO-NL ref. 6145833	Portugal, Azores, Sâo Miguel W	OQ241871—OQ241850—OQ222127
*G*. *tinctoria*	*G*. *tinctoria*	2019	NPPO-NL ref. 6145841	Portugal, Azores, Sâo Miguel W	OQ241872—OQ241851—OQ222128
*G*. *tinctoria*	*G*. *tinctoria*	2019	NPPO-NL ref. 6145851	Portugal, Azores, Sâo Miguel W	OQ241873—OQ241852—OQ222129
*G*. *tinctoria*	*G*. *tinctoria*	2019	NPPO-NL ref. 6145868	Portugal, Azores, Sâo Miguel W	OQ241874—OQ241853—OQ222130
*G*. *tinctoria*	*G*. *tinctoria*	2020	WAGPD: Vásquez-García s.n.	Chile W	OQ241866—OQ241845—OQ222122

^1^Species categorisation is based on molecular identification

^**2**^Origin: C = cultivated, W = wild, T = trade

Our barcoding project started in 2017 with samples from the Hortus Botanicus Leiden (Wieringa 9039, 9042) using silica dried material. Subsequently, plants were collected in the invasive range in New Zealand in February 2018 (Valkenburg 3913, 3915, 3928 silica dried) and in Ireland in September 2018 (Osborne s.n. silica dried). Plants in the horticultural trade in the Netherlands were collected in July 2018 (Valkenburg 3948, 3949 silica dried) and from the Azores in February 2019 (Valkenburg 4432 fresh). This was supplemented by plant material collected both in gardens and natural areas on Sâo Miquel Island, in the Azores (Osborne s.n.; 8 specimens silica dried) in September 2019. Fresh material was also obtained from the botanical gardens in Meise (Belgium) and in Paris (France) in November and December 2018. Additional leaf material (silica dried) and young plants were obtained from Stockholm University in December 2018 (Valkenburg 4433).

A sample from the native range in Chile was obtained in January 2020 (Vasquez-Garcia s.n. silica dried).

Additional measurement on leaves and inflorescence from *Gunnera* plants in the Hortus Botanicus Leiden were made in July 2022, to match molecular findings with morphological features. For each plant 5 leaves and 4 inflorescences, 10 branches of each inflorescence at midsection of the inflorescence, were used (1 *G*. *tinctoria*, 2 *G*. *“manicata*”).

Additional information regarding the ethical, cultural, and scientific considerations specific to inclusivity in global research is included in the S1 File.

### DNA extractions

Approximately 1 gram of plant material was ground in 5 ml GH+ grinding buffer (6 M guanidine hydrochloride, 0.2 M sodium acetate pH 5.2, 25 mM EDTA, and 2.5% PVP-10). Genomic DNA was isolated from 75 μl of the homogenate with the DNeasy plant mini kit (Qiagen, Venlo, The Netherlands) using 50 μL prewarmed (65°C) AE buffer for elution. DNA was stored at −20°C until used.

### PCR analyses

The PCR reactions for the chloroplast rbcL gene, the trnH-psbA intergenic spacer and nuclear ITS (partial 18S, ITS1, 5.8S, ITS2, partial 28S) loci were performed in 25 μL reaction mixes containing 200 nM of either primers rbcL-a F and rbcLa SI_Rev, trnH2 and psbAF or ITS5 and ITS4 (Table 2) respectively, 1 x MyFiTM Mix (Bio-line, Taunton, USA) and 2 ul genomic DNA. The cycle conditions for rbcL and trnH-psbA loci were as follows: 5 min at 95°C, followed by 5 cycles of 30 s at 94°C, 30 s at 45°C, 30 s at 72°C and 35 cycles of 30 s at 94°C, 30 s at 50°C, 30 s at 72°C and a final extension for 10 min at 72°C. The cycle condition for the ITS locus was as follows: 5 min at 95°C, followed by 40 cycles of 30 s at 94°C, 30 s at 52°C, 100 s at 72°C and a final extension for 10 m in at 72°C.

**Table 2:** Primers used in this study.

loci	Primer name	Primer sequence	Reference
rbcL	rbcL-a F	ATGTCACCACAAACAGAGACTAAAGC	[14]
	rbcLa SI_Rev	GTAAAATCAAGTCCACCRCG	[15]
trnH-psbA	trnH2	CGCGCATGGTGGATTCACAATCC	[16]
	psbAF	GTTATGCATGAACGTAATGCTC	[17]
ITS	ITS5	GGAAGTAAAAGTCGTAACAAGG	[18]
	ITS4	TCCTCCGCTTATTGATATGC	[18]

### Sanger sequencing

Two μl of ExoSAP-IT Express (Thermo Fisher Scientific, Bleiswijk, the Netherlands) was added to 5 μl PCR product and incubated (4 min 37°C, 1 min 80°C, 1 min 20°C) preceding bidirectional cycle sequencing with the BigDye Terminator v1.1 Cycle Sequencing Kit (Thermo Fisher Scientific, Bleiswijk, the Netherlands) using amplification primers as sequencing primers in separate reactions according to the manufacturer’s instructions. Cycle sequence products were purified with the DyeEx 2.0 Spin Kit (Qiagen,Venlo, the Netherlands) and sequenced using a 3500 Genetic Analyzer (Thermo Fisher Scientific, Bleiswijk, the Netherlands). Consensus sequences were generated from an assembly with trace files from both Sanger sequencing runs in Geneious Prime (Biomatters Auckland, New Zealand). Amplification primer sequences were trimmed in the assembly and, when needed, additional trimming was performed to obtain high-quality (PHRED > 30) consensus sequences.

## Results and discussion

### Morphological characteristics of plants in cultivation

The perceived morphological distinction between the two *Gunnera* species encountered in western Europe, as described in detail by Shaw et al. [19], was examined using measurements on plants in the Hortus Botanicus at Leiden, that were also sampled for our molecular work (Table 3). The inflorescence in *G*. *tinctoria* is of more modest dimensions, based on total length, length of the peduncle and diameter. Likewise, the leaves are of more modest dimensions. The partial inflorescences in *G*. *tinctoria* are shorter but have a larger diameter. These findings are in line with the comparative table reported in Shaw et al. [19]

**Table 3:** Morphology of living plants at the Hortus Botanicus Leiden; one plant of *G*. *tinctoria* (1), and two plants of “*G*. *manicata*” (2, 3). Each plant 5 leaves and 4 inflorescences, 10 branches of each inflorescence at midsection of inflorescence.

	Length inflorescence	Diameter inflorescence	Length peduncle	Length branch inflorescence	Diameter branch inflorescence	Length petiole	Length /width leaf
1	19	7.8	5	1–3 (2.3)	0.7–0.9 (0.81)	43	66/68.5
1	38.6	13	4	2–5 (3.3)	0.7–1.1 (0.88)	51.5	61/75
1	34	9	3	2–5 (3.4)	0.7–1 (0,81)	52	63/59
1	36	14	5.5	2–5 (2.8)	0.7–1.1(0.89)	41	61/64
1						29.5	18.5/23
2	65	17	13	1–8 (3.8)	0.3–0.6 (0.4)	92	73/103
2	63	21	17	2–7 (3.8)	0.2–0.5 (0.37)	137	73/104
2	59	16	21	1–9 (5.2)	0.3–0.5 (0.38)	133	53/79.5
2	69	14.5	19	1–8 (4.4)	0.3–0.6 (0.4)	138	79/107
2						66	59/88
3	76	19	23	2–9 (5.6)	0.3–0.6 (0.46)	151	117/182
3	84	24	19	1–12 (6.5)	0.3–0.6 (0.41)	135	87/138
3	43	25	22	5–11 (7.9)	0.3–0.6 (0.42)	122	74/94
3	86	25	20	2–12.5 (7.3)	0.3–0.7 (0.48)	118	94/119
3						154	86/128

Concerning the nomenclature issue for the proper naming of *G*. *manicata* and the resulting hybrid with *G*. *tinctoria* we refer to Shaw et al. [19]. The plants in cultivation in western Europe and found in New Zealand that are considered to be *G*. *manicata* are in fact hybrids and should be named *G*. *x cryptica* J.M.H.Shaw.

The *Gunnera* plants in the horticultural trade in Belgium were consistently mislabelled as *G*. *manicata*, whereas images of the flowering plants clearly identified them as *G*. *tinctoria* [5]. Likewise, *Gunnera* plants in the horticultural trade in the Netherlands are likely to have been mislabelled either accidentally or on purpose.

### Molecular characterisation of the plants in cultivation

The putative G. *manicata* and *G*. *tinctoria* material collected from the Hortus Botanicus Leiden had identical rbcL sequences and differed only 1 nt in their trnH-psbA spacer sequence (Table 4). Blast searches with the trnh-psbA spacer sequence at NCBI GenBank showed that there was 99.7% identity overlap with a *G*. *chilensis* Lam. (synonym of *G*. *tinctoria*) accession (AB250752), with no *G*. *manicata* trnH-psbA sequences present in NCBI GenBank at the time. As the inter-specific variation of the trnH-psbA loci is often large enough for species identification, it was assumed that the specimen labelled as *G*. *manicata* from the Hortus Botanicus Leiden was also *G*. *tinctoria*.

**Table 4:** Nucleotide differences between *G*. *tinctoria*, *G*. *manicata* and their hybrids for the trnH-psbA spacer en ITS loci.

loci	trnH-psbA	ITS										
nt position	300	93	111	191	250	266	271	272	298	407	512	671
*G*. *tinctoria*	A	A	A	G	T	G	A	A	C	T	A	A
*G*. *manicata*	G	G	T	C	C	A	-	-	T	C	G	G
*G*. *tinctoria x G*. *manicata*	G	R	W	S	Y	R	N^1^	N	Y	Y	R	R
*G*. *manicata x G*. *tinctoria*	A	R	W	S	Y	R	N	N	Y	Y	R	R

^1^N means the nucleotide A or no nucleotide (-)

The *G*. *tinctoria* plants collected in New Zealand contained similar cpDNA markers. However, plants in the horticultural trade sampled in July 2018 did give contrasting results as a plant resembling a depauperate *G*. *manicata* (Valkenburg 3948) was identified as *G*. *tinctoria* based on their cpDNA.

Several *Gunnera* spp sequences have been published recently [20], which revealed that there is very small variation (1–5 nt) in the trnH-psbA sequences among the species originating from South America, including *G*. *brephogea* Linden & André, *G*. *boliviana* Morong, *G*. *chilensis (*synonym of *G*. *tinctoria)*, *G*. *kauaiensis* Rock, *G*. *manicata*, *G peltata* Phil. and *G*. *petaloidea* Gaudich. The trnH-psbA sequence for *G*. *manicata* (MH017175) was 100% identical to that obtained from material labelled as *G*. *manicata* from the Hortus Botanicus Leiden.

In addition to the cpDNA markers we also included ITS for our analysis as Bacon et al. [20] has published several ITS sequences for *Gunnera* species, which show more variation than the use of trnH-psbA markers. To obtain ‘true’ *G*. *manicata* we contacted Stockholm University to verify the presence of living material of the herbarium voucher from the Wanntorp 560 collection [11]. Unfortunately, the herbarium specimen could not be retrieved, nor could any living material that was specifically labelled as Wanntorp 560 *G*. *manicata*. However, plants labelled as *G*. *manicata*” Klon B” were present in the Department’s greenhouse collection. This material (Valkenburg 4433) gave a 100% match with the GenBank data for Wanntrop 560 (trnH-psbA (MH017175) [20] and ITS (AF447740) [11]) and a collection from Brazil (Pouso Redondo, Serra do Matador, 28 Dec 2000),A. Reis s.n. (ITS AF447741 [11]). Young plants transferred from Stockholm to the greenhouse at Wageningen exhibited poor growth and died after six months.

All previous samples were then reanalysed and new samples from botanical gardens in Belgium, France and the Azores, as well as wild material collected from Chile and the Azores were included. Of the plants labelled as *G*. *manicata* all were found to be hybrids between *G*. *manicata* and *G*. *tinctoria* with *G*. *manicata* being the mother plant, as ambiguous nucleotides were found at the position where the ITS sequences of *G*. *manicata* and *G tinctoria* differ, while the trnH-psbA sequence was identical to that of *G*. *manicata* (see Tables 1 and 4). All plants labelled as *G*. *tinctoria* from gardens in the Netherlands or material obtained from naturalised or native provenances in Ireland, the Azores, New Zealand and Chile were 100% pure *G*. *tinctoria*.

A plant labelled *G*. *manicata* that was identified from the horticultural trade (Valkenburg 3948), which had a slightly more condensed inflorescence, was also found to be of hybrid origin but with *G*. *tinctoria* as the mother plant. Another plant labelled as *G*. *manicata* (Valkenburg 4432) was simply mislabelled and turned out to be pure *G*. *tinctoria*.

Clearly, the earlier statement that *G*. *tinctoria* does not form viable hybrids [12] is incorrect. An earlier report by Palkovic [21] also reported the occurrence of hybrids between two larg*e Gunnera* species, *G*. *insignis* (Oerst.) Oerst. and *G*. *talamancana* H.Weber & L.E.Mora in Costa Rica, indicating that hybridisation within the genus may be more common than once thought.

Aside from the common problem of misidentification of plant material this study also raises some interesting questions about the identification and origin of the hybrid material. If all the material in gardens or circulating in the horticultural trade that is labelled a*s G*. *manicata* are hybrids, where did this material come from and when did the hybridisation occur? Given that our results indicate that all naturalised plants are pure *G*. *tinctoria*, were any ‘pure’ *G*. *manicata* plants ever introduced?

The status of hybrids in the context of the EU regulation 1143/2014 and the associated delegated act is somewhat ambiguous. Whereas, the EU regulation 1143/2014 defines an alien species under article 3(1) as “any live specimen of a species, subspecies or lower taxon of animals, plants, fungi or micro-organisms introduced outside its natural range; it includes any part, gametes, seeds, eggs or propagules of such species, as well as any hybrids, varieties or breeds that might survive and subsequently reproduce;” The associated delegated act clearly specifies under article 5 (1)(a)(2) “It shall be clearly stated if the risk assessment covers more than one species, or if it excludes or only includes certain subspecies, lower taxa, hybrids, varieties or breeds (and if so, which subspecies, lower taxa, hybrids, varieties or breeds). Any such choice must be properly justified.” For the *G*. *tinctoria* hybrids, there is no evidence that these are invasive and persist outside of cultivation, as all the naturalised material was identified as *G*. *tinctoria*. Based on this information the legislation may need to be revised to account for scenarios where hybrids are less problematic than their parents.

## Conclusion

Based on the evidence from this study all plants found in gardens in the Netherlands, Belgium, France and New Zealand and labelled as *G*. *manicata* are likely of hybrid origin with *G*. *manicata* as the mother plant. In the horticultural trade most plants labelled as *G*. *manicata*, are likewise hybrids that have *G*. *manicata* as the mother plant. However, some of the plants in the horticultural trade that are labelled as *G*. *manicata* and have a slightly more condensed inflorescence, may have *G*. *tinctoria* as the mother plant. Confirmation of the extent of hybridisation and the parental contributions will, however, require a more extensive study using a wider range of genotypes. Clearly, several other *G*. *tinctoria* plants, including naturalised material, have simply been mislabelled as *G*. *manicata*. All plants labelled as *G*. *tinctoria* and found either in gardens or as naturalised provenances are 100% pure *G*. *tinctoria*.

## Supporting information

S1 File Inclusivity in global research. (DOCX)

## References

[1] Brunel S. Pathway analysis: aquatic plants imported in 10 EPPO countries. Bull OEPP 2009;39: 201–213.

[2] Hulme PE, Brundu G, Carboni M, Dehnen-Schmutz K, Dullinger S, Early R et al. Integrating invasive species policies across ornamental horticulture supply chains to prevent plant invasions. J Appl Ecol 2018;55: 92–98. doi: 10.1111/1365-2664.12953

[3] Thum RA, Mercer AT, Wcisel DJ. Loopholes in the regulation of invasive species: genetic identifications identify mislabelling of prohibited aquarium plants. Biol Invasions 2012;14: 929–937. doi: 10.1007/s10530-011-0130-8

[4] Verbrugge LNH, Leuven RSEW, van Valkenburg JLCH, van den Born RJG. Evaluating stakeholder awareness and involvement in risk prevention of aquatic invasive plant species by a national code of conduct. Aquat Invasions 2014;9: 369–381.

[5] Van den Neucker T, Scheers K. Mislabelling may explain why some prohibited invasive plants are still being sold in Belgium. Knowl Manag Aquat Ecosyst 2022;423, 8. 10.1051/kmae/2022005.

[6] Zaya DN, Leicht-Young SA, Pavlovic NB, Hetrea CS, Ashley MV. Mislabelling of an invasive vine (*Celastrus orbiculatus*) as a native congener (C. scandens) in horticulture. Invasive Plant Sci Manag 2017;10: 313–321. doi: 10.1017/inp.2017.37

[7] Wanntorp l, Wanntorp H-E, Oxelman B, Källersjö M. Phylogeny of *Gunnera*. Plant Syst Evol 2001;226(1–2):85–107. doi: 10.1007/s006060170075

[8] Wanntorp l, Wanntorp H-E, Rutishauser R. On the homology of the scales in *Gunnera* (Gunneraceae) Bot J Linn Soc 2003;142(3): 301–308. doi: 10.1046/j.1095-8339.2003.00185.x

[9] Clement EJ. What is *Gunnera manicata*?–and whence? B.S.B.I. News 2003; 93: 52–55.

[10] Wanntorp L. Origin and identity of the cultivated *Gunnera manicata*. The Plantsman n.s. 2003;2(4): 221–225.

[11] Wanntorp L, Wanntorp H-E, Källersjö M. The identity of *Gunnera manicata* Linden ex André –resolving a Brazilian-Colombian enigma. Taxon 2002;51: 493–497.

[12] Gioria M, Osborne BA. Biological Flora of the British Isles: *Gunnera tinctoria*. J Ecol 2013;101: 243–264. doi: 10.1111/1365-2745.12022

[13] Osborne B, Sprent JI. Ecology of the *Nostoc-Gunnera* symbiosis. In: Rai AN, Bergman B, Rasmussen U, editors. Cyanobacteria in Symbiosis. Dordrecht: Kluwer Academic Publishers; 2002. pp. 233–251.

[14] Kress WJ, Erickson DL. A Two-Locus Global DNA Barcode for Land Plants: The Coding rbcL Gene Complements the Non-Coding trnH-psbA Spacer Region. PLoS One 2007; 2(6): e508. doi: 10.1371/journal.pone.0000508 17551588 PMC1876818

[15] Kress WJ, Erickson DL, Jones FA, Swenson NG, Perez R, Sanjur O et al. Plant DNA barcodes and a community phylogeny of a tropical forest dynamics plot in Panama. Proc Natl Acad Sci U S A 2009; 106: 18621–18626. doi: 10.1073/pnas.0909820106 19841276 PMC2763884

[16] Tate JA. Systematics and evolution of *Tarasa* (Malvaceae): an enigmatic Andean polyploid genus. PHD Thesis. The University of Texas (Austin). 2002.

[17] Sang T, Crawford DJ, Stuessy TF. Chloroplast DNA phylogeny, reticulate evolution, and biogeography of *Paeonia* (Paeoniaceae). Am J Bot 1997; 84: 1120–1136. 21708667

[18] White TJ, Bruns T, Lee S, Taylor J. Amplification and direct sequencing of fungal ribosomal RNA genes for phylogenetics. In: Innis MA, Gelfand DH, Sninsky JJ, White TJeditors. PCR Protocols: A guide to methods and applications. Academic Press, San Diego; 1990. pp. 315–322.

[19] Shaw JMH, Edwards D, David J. A new spontaneous hybrid in *Gunnera* subgenus *Panke* (Gunneraceae) widespread in the British Isles, with notes on the typification of *G*. *manicata*. Br Ir Bot 2022;4(3): 364–384.

[20] Bacon CD, Velásquez-Puentes FJ, Hinojosa LF, Schwartz T, Oxelman B, Pfeil B et al. Evolutionary persistence in *Gunnera* and the contribution of southern plant groups to the tropical Andes biodiversity hotspot. PeerJ 2018;6:e4388 10.7717/peerj.4388. 29576938 PMC5858603

[21] Palkovic LA. A hybrid of *Gunnera* from Costa Rica. Syst Bot 1978;3: 228–235.

